# To the Editors of the Medical and Physical Journal

**Published:** 1801-02

**Authors:** Charles Collier

**Affiliations:** Aylesbury Street, Clerkenwell


### *To the Editors of the Medical and Phyfical Journal.*

GENTLEMEN,

ABOUT feven o'clock on Wednefday evening, (Dec. 31) a hurrying meffage was brought, requiring Mr. Chamberlaine's attendance to a young man who had fwallowed, with an intention of putting an end to himfelf, a quantity of laudanum.

Mr. Chamberlaine being at that time at fome diftance, I offered my fervices, which were accepted; and knowing no time muft be loft, I took with me ʒfs. of ipecacuanha, and fix grains of tartar emetic. I found the young man in a ftate of infenfibility, the pupils of the eyes widely dilated; pulfe irregular, and fcarcely perceptible; fubfultus tendinum; ftertor; cold fweats, and every bad fymptom.

A phial which, when full, might contain an ounce and a half of laudanum, was produced. On inquiry, I learned that he had brought it home quite full; he had taken the largeft half, or rather more than fix drachms: and from every circumftance that could be collected, a fpace of time not lefs than fifteen, or perhaps, even twenty minutes, muft have elapfed before it was difcovered that he had taken it; and fome minutes more elapfed, elapfed, of courfe, while affiftance was fought after. The emetic I brought with me I mixed in fome warm water, and his mouth being opened with fome difficulty, it was poured in; but finding that this produced only two convulfive motions of the ftomach, I haftened home, (ordering the people with him to keep him in continual motion, and on no account whatever to fuffer fleep to come on) and returned with two papers, the one containing ℈fs. and the other ℈j. of zincum vitriolatum, with a fmall quantity of tartar emetic: I gave him the former one directly, and the latter in about four minutes after, drenching him with great quantities of warm water. This rouzed him a little; but ftill, as it only vomitted him twice, and that not in fuch a manner as I could depend upon for the prefervation of his life, I again went home, and returned with two papers containing ℈j. in each. I now found convulfions and ftupor coming on very quick, in a greater degree than at firft, with his teeth clafped, and pulfe ftill more weak and irregular; I forced open his mouth, and poured down the folution of zincum vitriolatum, continuing to give him plenty of warm water, and followed up this quantity with the fecond; in about five minutes thefe vomitted him twice very well, and on my repeating the warm water, in which I diffolved ℈fs. of zincum vitriolatum, they vomitted him in fuch manner, as to put him out of all DANGER. The convulfive motions of the upper extremities now began to go off by degrees, and every other dangerous and alarming fymptom, peculiar to this narcotic drug, to fubfide. I ordered him to be walked about the room at intervals, and to be kept awake for upwards of fix hours. He felt his ftomach and throat very rigid and fore the enfuing morning, but when I went in the evening, accompanied by Mr. Chamberlaine, he was perfectly recovered; and on vifiting him fince then, found him perfectly well and very thankful. I am, &c.

*Aylefbury Street, Clerkenwell,*

*Jan.* 5, 1801.

CHARLES COLLIER.

---

